# RNA-seq data for olive flounder (Paralichthys olivaceus) according to water temperature

**DOI:** 10.1016/j.dib.2019.104384

**Published:** 2019-08-12

**Authors:** Dong-Hee Cho, Chan-Il Park

**Affiliations:** Institute of Marine Industry, College of Marine Science, Gyeongsang National University, 455, Tongyeong 650-160, Republic of Korea

**Keywords:** RNA-seq data, Olive flounder, Water temperature stimulation

## Abstract

We provide raw data from a transcriptomic analysis of olive flounder in response to changes in water temperature. At the time of this analysis, the olive flounder genome was not yet available in China, and there were no related references. Therefore, assembly was carried out using the de novo method to reveal the entire nucleotide sequence based on the nucleotide sequence information of the sequenced reads. The functions of expressed genes based on Gene Ontology analysis are also categorized and presented.

**Table:** Specifications Table

Subject area	Biology
More specific subject area	Evolutionary and Reproductive Biology, Transcriptomics
Type of data	Transcriptomics (RNA-seq)
How data was acquired	High-throughput sequencing (Illumina HiScanSQ)
Data format	Raw data
Experimental factors	RNA-seq data with time-varying at 20 °C water temperature and 20 ° C-13 °C low-temperature stimulation groups.
Experimental features	RNA-seq data obtained from the analysis of transcripts of the two groups were compared and analysed.
Data source location	Gyeongsang National University, Tongyeong, Republic of Korea
Data accessibility	Data is available in the article and at: https://www.ncbi.nlm.nih.gov/bioproject/552408https://www.ncbi.nlm.nih.gov/pubmed/18957448

**Table:** 

**Value of the data** • Transcriptome data for olive flounder can provide insight into the gene expression alterations in this species in response to changes in water temperature and can further provide insights into other fish species. • Comparison of gene expression data between low and high temperatures reveals a preliminary stress-related gene associated with environmental changes. • Functional analysis data can be used in future studies to anticipate the biological pathways of olive flounder when the water temperature suddenly changes.

## 1 Data

The major causes of stress in aquaculture can be classified into chemical and physical factors. Among the physical factors, in particular, water temperature changes cause stress to fish and affect physiological activity. Excessive temperature stimulation also causes mortality. In addition, sudden changes in water temperature due to the cold water in the East Sea of Korea during the summer may slow fish growth and cause disease. These data show RNA-seq results of the olive flounder, a major marine aquaculture species in Korea, as a function of water temperature. The results of sequence quality assessment for the whole sample are summarized in Table 1 as the number of reads and the average base pairs (bp). The total sequence length was 121,120,858 bp; the number of unigenes was 108,151; and the average length of the unigenes was 1,120 bp. The mapped reads were normalized to show the amount of RNA expressed as Fragments Per Kilobase of transcript per Million mapped reads (FPKM) (Supplementary Table 1). The number of reads mapped through RNA-seq can be used to determine the expression level of each sample by gene or transcript. However, the sequencing data size may differ for each sample, making it difficult to define the expression amount as the number of mapped reads. Thus, this value cannot be viewed as objective, since the number of mapped reads varies with the length of a gene or a transcript.

**Table 1:** Overview of basic quality control metrics using FastQC.

No	Name	Raw			Clean		Low Quality reads
		Read	Base	Base (>Q30)	Reads	Basepair	
1	13_4h-1	69,319,080	7,001,227,080	6,484,523,896	62,814,494 (90.6%)	6,331,352,144 (90.4%)	5,715,436 (8.2%)
2	13_4h-2	59,557,470	6,015,304,470	5,567,692,564	53,893,806 (90.5%)	5,432,185,850 (90.3%)	4,962,482 (8.3%)
3	13_4h-3	49,882,156	5,038,097,756	4,719,469,928	45,595,286 (91.4%)	4,596,728,403 (91.2%)	3,855,530 (7.7%)
4	20_4h-1	48,262,836	4,874,546,436	4,528,745,824	43,968,802 (91.1%)	4,431,911,414 (90.9%)	3,735,882 (7.7%)
5	20_4h-2	47,430,868	4,790,517,668	4,485,849,273	43,555,772 (91.8%)	4,390,745,375 (91.7%)	3,449,314 (7.3%)
6	20_4h-3	50,348,520	5,085,200,520	4,722,910,471	45,883,438 (91.1%)	4,624,870,863 (90.9%)	3,889,886 (7.7%)
7	13_1d-1	42,541,146	4,296,655,746	4,129,116,384	40,282,548 (94.7%)	4,055,354,699 (94.4%)	2,046,620 (4.8%)
8	13_1d-2	47,093,194	4,756,412,594	4,575,372,410	44,637,738 (94.8%)	4,476,852,015 (94.1%)	2,015,274 (4.3%)
9	13_1d-3	61,440,238	6,205,464,038	5,981,339,423	58,570,126 (95.3%)	5,843,501,636 (94.2%)	2,620,912 (4.3%)
10	13_3d-1	55,446,104	5,600,056,504	5,385,762,197	52,601,604 (94.9%)	5,296,121,238 (94.6%)	2,627,672 (4.7%)
11	13_3d-2	67,974,814	6,865,456,214	6,560,828,225	66,018,672 (97.1%)	6,614,958,347 (96.4%)	1,327,800 (2.0%)
12	13_3d-3	72,223,672	7,294,590,872	7,022,733,057	68,835,242 (95.3%)	6,842,324,713 (93.8%)	3,060,482 (4.2%)
13	13_7d-1	48,892,048	4,938,096,848	4,738,673,248	46,241,178 (94.6%)	4,655,618,990 (94.3%)	2,423,010 (5.0%)
14	13_7d-2	62,583,924	6,320,976,324	6,102,495,760	59,843,272 (95.6%)	6,002,023,301 (95.0%)	2,589,246 (4.1%)
15	13_7d-3	64,294,090	6,493,703,090	6,269,626,145	61,317,032 (95.4%)	6,096,617,632 (93.9%)	2,405,366 (3.7%)
16	20_1d-1	54,143,238	5,468,467,038	5,222,264,253	50,900,060 (94.0%)	5,129,435,815 (93.8%)	3,124,008 (5.8%)
17	20_1d-2	70,613,706	7,131,984,306	6,806,315,910	68,505,362 (97.0%)	6,882,716,586 (96.5%)	1,440,928 (2.0%)
18	20_1d-3	64,486,842	6,513,171,042	6,219,048,374	62,591,644 (97.1%)	6,285,912,170 (96.5%)	1,273,076 (2.0%)
19	20_3d-1	58,029,188	5,860,947,988	5,549,838,146	53,735,482 (92.6%)	5,413,516,607 (92.4%)	4,208,126 (7.3%)
20	20_3d-2	67,456,288	6,813,085,088	6,499,955,352	65,387,018 (96.9%)	6,566,378,315 (96.4%)	1,425,472 (2.1%)
21	20_3d-3	67,763,052	6,844,068,252	6,527,403,387	65,775,416 (97.1%)	6,620,388,638 (96.7%)	1,421,108 (2.1%)
22	20_7d-1	50,062,484	5,056,310,884	4,840,809,726	47,337,722 (94.6%)	4,769,786,141 (94.3%)	2,664,910 (5.3%)
23	20_7d-2	57,332,324	5,790,564,724	5,516,838,571	55,546,208 (96.9%)	5,590,763,436 (96.5%)	1,294,546 (2.3%)
24	20_7d-3	61,758,646	6,237,623,246	5,949,819,820	59,798,930 (96.8%)	6,018,724,469 (96.5%)	1,386,490 (2.2%)

Therefore, normalization of differential gene expression is required to reduce error and obtain a more objective value. One of the popular methods to do so is the FPKM calculation method; FPKM is calculated using the number of fragments per transcript. For a paired-end read, a pair of reads constitutes a single fragment; therefore, FPKM can be used for RNA-seq analysis of paired-end reads. The values for the expression of these genes were found to be more than 1 and are shown separately in Table 2. Table 3 shows the number of genes exhibiting a p-value less than 0.05 and a greater than two-fold difference in their expression level based on the analysis of differentially expressed genes (DEGs) between the 13 °C and 20 °C groups for each time period. The Gene ID, p-value, log2fc value, etc., for each section are attached to Supplementary Table 2. Genes with a p-value of less than 0.001 in the DEG analysis were divided into three independent categories: Molecular Function, Biological Process, and Cellular Component, through Gene Ontology (GO) analysis (Table 4). Detailed GO IDs, categories, gene names, descriptions, etc., are provided in Supplementary Table 3.

**Table 2:** Overview of gene expression.

Name	Gene			Gene (>fpkm 1.0)		
	Expressed	Known	Novel	Expressed	Known	Novel
13_4h-1	79,862	39,487	40,375	79,120	38,996	40,124
13_4h-2	75,737	37,658	38,079	75,246	37,333	37,913
13_4h-3	63,700	34,737	28,963	63,465	34,545	28,920
20_4h-1	66,230	35,528	30,702	66,017	35,348	30,669
20_4h-2	66,555	35,706	30,849	66,327	35,518	30,809
20_4h-3	67,076	35,777	31,299	66,854	35,602	31,252
13_1d-1	38,404	24,900	13,504	38,268	24,815	13,453
13_1d-2	32,998	23,337	9,661	32,909	23,272	9,637
13_1d-3	33,460	22,483	10,977	33,326	22,409	10,917
13_3d-1	39,876	25,369	14,507	39,718	25,274	14,444
13_3d-2	36,244	21,304	14,940	36,049	21,226	14,823
13_3d-3	39,215	24,407	14,808	38,969	24,302	14,667
13_7d-1	57,810	31,698	26,112	57,356	31,508	25,848
13_7d-2	48,119	27,662	20,457	47,831	27,554	20,277
13_7d-3	44,502	27,065	17,437	43,909	26,835	17,074
20_1d-1	33,843	21,127	12,716	33,732	21,061	12,671
20_1d-2	38,708	24,787	13,921	38,580	24,707	13,873
20_1d-3	35,155	24,050	11,105	35,036	23,961	11,075
20_3d-1	42,470	25,408	17,062	42,321	25,332	16,989
20_3d-2	47,302	28,720	18,582	47,083	28,601	18,482
20_3d-3	42,883	25,120	17,763	42,717	25,035	17,682
20_7d-1	53,467	29,100	24,367	53,202	28,981	24,221
20_7d-2	57,495	31,309	26,186	57,170	31,159	26,011
20_7d-3	50,974	28,823	22,151	50,731	28,720	22,011

**Table 3:** Overview of differentially expressed genes.

Group 1 (G1)	Group 2 (G2)	Genes		
		Sum*	Up (G2 only)**	Down (G1 only)***
13_4h-1,13_4h-2,13_4h-3	20_4h-1,20_4h-2,20_4h-3	4,273	1,214 (177)	3,059 (1,260)
13_1d-1,13_1d-2,13_1d-3	20_1d-1,20_1d-2,20_1d-3	2,079	887 (558)	1,192 (556)
13_3d-1,13_3d-2,13_3d-3	20_3d-1,20_3d-2,20_3d-3	1,912	1,251 (804)	661 (464)
13_7d-1,13_7d-2,13_7d-3	20_7d-1,20_7d-2,20_7d-3	3,434	1,686 (783)	1,748 (764)

* When the p-value of the expression level in the same gene between the two groups was less than 0.05, the difference was significant and the number was indicated.

The number of genes significantly higher in the G2 group than in the G1 group (**) and the number of low genes (***) are indicated.

**Table 4:** Overview of gene ontology.

DEGs Group	DEG*	GO	MF**	BP***	CC****
13_4h-1,13_4h-2,13_4h-3 vs 20_4h-1,20_4h-2,20_4h-3	2671	5160	11/1082 (1.0%)	28/3490 (0.8%)	13/588 (2.2%)
13_1d-1,13_1d-2,13_1d-3 vs 20_1d-1,20_1d-2,20_1d-3	1598	4200	11/942 (1.2%)	11/2759 (0.4%)	6/499 (1.2%)
13_3d-1,13_3d-2,13_3d-3 vs 20_3d-1,20_3d-2,20_3d-3	1465	3952	4/788 (0.5%)	7/2677 (0.3%)	0/487 (0.0%)
13_7d-1,13_7d-2,13_7d-3 vs 20_7d-1,20_7d-2,20_7d-3	2425	5133	11/1082 (1.0%)	21/3476 (0.6%)	8/575 (1.4%)

* Among the genes with significant difference between the two groups, the number of genes whose p-value is less than 0.001 is indicated.

** MF: Molecular function, *** BP: Biological process, **** CC: Cellular component.

## 2 Experimental design, materials, and methods

The average weight and total length of the olive flounders used in the study were 124.2 g and 23.76 cm, respectively. The fish were acclimated at 20 °C for one week. The experiments were divided into two groups: group 1, in which the water temperature was decreased to 13 °C within 30 minutes; and group 2, which was maintained at 20 °C. Sampling was performed three times, and samples named “water temperature_intermediate sampling time-number of repeats”. For example, the 13 °C cold stimulation, day 3, 2nd sample was named 13_4d-2. The kidneys of the fish can be classified into head, body, and tail. The head kidney is located at the front of the kidney (near the head of the fish) and is said to be involved in hematopoietic and hormonal secretion. Head kidney were sampled from each group at 4 hours and 1 day, 3 days, and 7 days. Total RNA was isolated from the sampled head kidneys using TRIzol (Invitrogen, Carlsbad, CA, USA). We created a dUTP second strand library starting from 200 ng. Following, we fragmented RNA in 1 × fragmentation buffer (Affymetrix) at 80 °C for 4 min, purified and concentrated the RNA to 6 μL after ethanol precipitation. We added an index (8-base barcode) to each library to enable pooling of these libraries. In addition, the adaptor ligation step was performed using 1.2 μL of index adaptor mix and 4,000 cohesive end units of T4 DNA Ligase (New England Biolabs) overnight at 16 °C in a final volume of 20 μL. Finally, we generated libraries with an insert size ranging from 225 to 425 bp. The sequenced raw data were assembled and cleaned by removing regions with low quality score using Quality trimming of FastQC program [1]. Here we implemented assembly using Trinity. Trinity is a method for efficient and powerful de novo reconstruction of transcriptomes consisting of three software modules: Inchworm, Chrysalis and Butterfly sequentially applied to handle large quantities of RNA-Seq readings [2]. We used the CD-HIT program to produce a non-redundant dataset through clustering and alignment of the sequencing data. Confirmation procedures and clustering procedures were used to support full parallel processing. CD-HIT was implemented in the C ++ programming language and uses OpenMP (http://www.openmp.org) for parallelization [3]. The RSEM software package was used to estimate the expression levels of genes and isoforms from RNA-seq data. Typical implementation of RSEM consists of two steps. First, a set of reference transcription sequences is generated and preprocessed for use by subsequent RSEM steps. Second, a series of RNA-Seq readings are aligned with the reference transcript and the resulting alignments are used to estimate the abundance and confidence intervals [4]. InterProScan and Blast2GO software were used to predict protein sequence domains and perform functional analysis. The InterPro database is available on the web server (http://www.ebi.ac.uk/interpro). The database can be searched using the query order or through the text search function. For complete proteomes, InterPro results are available on the Integr8 Proteome Analysis page (http://www.ebi.ac.uk/integr8) [5], [6].

## Appendix A Supplementary data

Multimedia component 2 Multimedia component 2

Multimedia component 3 t Multimedia component 3

## References

[1] S. Andrews, FastQC: a quality control tool for high throughput sequence data, Available online at: http://www.bioinformatics.babraham.ac.uk/projects/fastqc/.

[2] Grabherr M.G., Haas B.J., Yassour M., Levin J.Z., Thompson D.A., Amit I., Adiconis X., Fan L., Raychowdhury R., Zeng Q., Chen Z., Mauceli E., Hacohen N., Gnirke A., Rhind N., di Palma F., Birren B.W., Nusbaum C., Lindblad-Toh K., Friedman N., Regev A. (2011). Full-length transcriptome assembly from RNA-seq data without a reference genome. Nat. Biotechnol..

[3] Fu L., Niu B., Zhu Z., Wu S., Li W., CD-HIT (2012). Accelerated for clustering the next-generation sequencing data. Bioinformatics.

[4] Li B., Dewey C.N. (2011). RSEM: accurate transcript quantification from RNA-Seq data with or without a reference genome. BMC Bioinf..

[5] Mulder N., Apweiler R. (2007). InterPro and InterProScan: tools for protein sequence classification and comparison. Methods Mol. Biol..

[6] Barrell D., Dimmer E., Huntley R.P., Binns D., O'Donovan C., Apweiler R. (2009). The GOA database in 2009 an integrated Gene Ontology Annotation resource. Nucleic Acids Res..

